# Postoperative statin treatment may be associated with improved mortality in patients with myocardial injury after noncardiac surgery

**DOI:** 10.1038/s41598-020-68511-3

**Published:** 2020-07-15

**Authors:** Jungchan Park, Jihoon Kim, Seung-Hwa Lee, Jong Hwan Lee, Jeong Jin Min, Ji-hye Kwon, Ah Ran Oh, Wonho Seo, Cheol Won Hyeon, Kwangmo Yang, Jin-ho Choi, Sang-Chol Lee, Kyunga Kim, Joonghyun Ahn, Hyeon‐Cheol Gwon

**Affiliations:** 1 Department of Anesthesiology and Pain Medicine, Samsung Medical Center, Sungkyunkwan University School of Medicine, Seoul, South Korea; 2 Division of Cardiology, Department of Medicine, Heart Vascular Stroke Institute, Samsung Medical Center, Sungkyunkwan University School of Medicine, 81 Irwon-ro, Gangnam-gu, Seoul, South Korea; 3 Centers for Health Promotion, Samsung Medical Center, Sungkyunkwan University School of Medicine, Seoul, South Korea; 4 Statistics and Data Center, Research Institute for Future Medicine, Samsung Medical Center, Seoul, South Korea; 5 Department of Digital Health, SAIHST, Sungkyunkwan University, Seoul, South Korea

**Keywords:** Biomarkers, Medical research, Risk factors

## Abstract

Myocardial injury after noncardiac surgery (MINS) is recently accepted as a strong predictor of mortality, regardless of symptoms. However, anticoagulation is the only established treatment. This study aimed to evaluate the association between statin treatment and mortality after MINS. From January 2010 to June 2019, a total of 5,267 adult patients who were discharged after the occurrence of MINS were enrolled. The patients were divided into two groups according to statin prescription at discharge. The outcomes were 1-year and overall mortalities. Of the total 5,109 patients, 1,331 (26.1%) patients were in the statin group and 3,778 (73.9%) patients were in the no statin group. The 1-year and overall mortalities were significantly lower in the statin group compared with the no statin group (6.1% vs. 13.3%; hazard ratio [HR], 0.55; 95% confidence interval [CI], 0.41–0.74; p < 0.001 for 1-year mortality and 15.0% vs. 25.0%; HR, 0.62; 95% CI, 0.51–0.76; p < 0.001 for overall mortality). Analyses after inverse probability treatment weighting showed similar results (HR, 0.61; 95% CI, 0.50–0.74; p < 0.001 for 1-year mortality and HR, 0.70; 95% CI, 0.54–0.90; p = 0.006 for overall mortality), and the mortalities did not differ according to the dose of statin. Our results suggest that statin treatment may be associated with improved survival after MINS. A trial is needed to confirm this finding and establish causality.

## Introduction

Myocardial injury after noncardiac surgery (MINS), regardless of ischemic symptoms, showed an independent association with cardiovascular events and mortality up to the first 2 years after surgery^1,2^. However, it was only within the last 5 years that MINS was recognized as a strong predictor of mortality; before then, perioperative management has been focused on preventing symptomatic myocardial infarction^3^. Therefore, treatment for the patients with MINS has not been fully established.

Statin is included as an optimal therapy for prevention and treatment of myocardial infarction mostly based on findings from nonoperative settings^4,5^. In addition to lipid-lowering effects, statins exhibit numerous protective effects on the cardiovascular system including improved endothelial function, enhanced stability of atherosclerotic plaques, and decreased oxidative stress and inflammation^6,7^. By attenuating inflammation and oxidative stress during the perioperative period, statin has also been shown to decrease operative complications that are not cardiac origin^8^. In addition, it is one of the few cardiovascular medications that were demonstrated to actually improve the outcome of patients with myocardial injury in observational studies^3^. However, the benefit of statin during the perioperative period remains controversial, because some randomized trials failed to demonstrate the primary prevention of postoperative cardiovascular complications including MINS, especially in patients with low cardiac risk^9,10^. Instead, another randomized trial showed a protective effect of statin use in patients, limited to those who had atherosclerotic coronary disease and received long-term therapy after surgery^11^. Based on these previous results, we hypothesized that in patients who were diagnosed with MINS, statin prescription at discharge may improve long-term mortalities.

## Results

**Figure 1:**
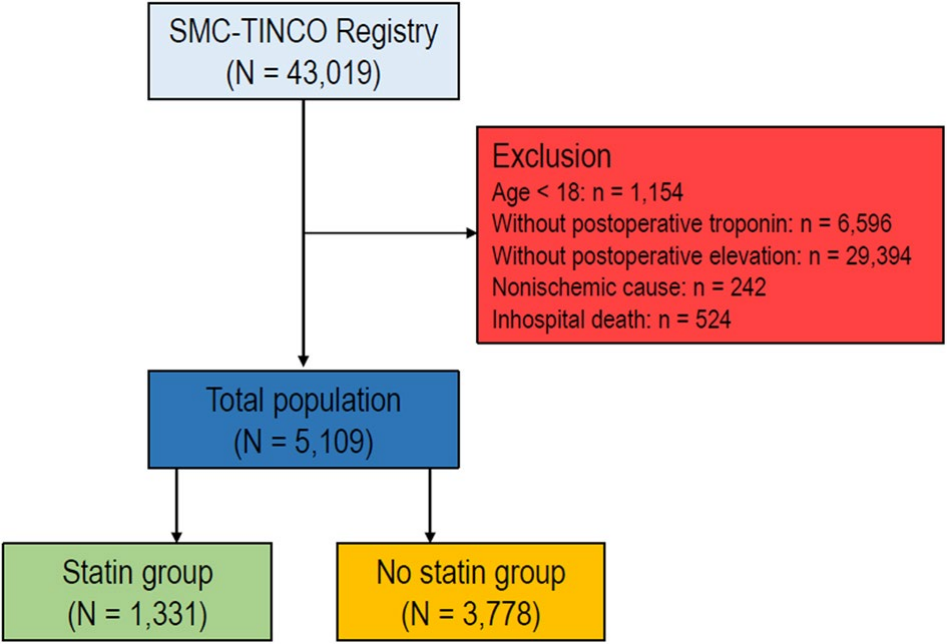
The flowchart of patients.

We excluded the following patients for this study: (1) 1,154 patients who were younger than 18 years at the time of surgery, (2) 6,596 patients without postoperative hs-cTn I, (3) 29,394 patients without postoperative hs-cTn elevation, (4) 242 patients who had cardiac massage before the diagnosis of MINS, and (5) 524 patients with mortality during the hospital stay. Finally a total of 5,109 patients was divided into two groups according to statin prescription at discharge: 3,778 (73.9%) patients were in the no statin group and 1,331 (26.1%) patients were in the statin group. The flowchart of the patients is summarized in Fig. 1. In this study, we enrolled 5,109 adult patients who were discharged alive after suffering myocardial injury after all types of noncardiac surgery that was performed at our institution during the study period and compared mortalities within 1-year after discharge and overall mortalities during median follow-up period of 711 days (IQR 223–1559).

The median duration from surgery to the peak postoperative hs-cTn I level was postoperative one day (IQR 1–3). The median duration from surgery to postoperative statin use was one day (IQR 1–4). The levels of low-density lipoprotein at discharge were 79.7 mg/dL in the no statin group 77.9 mg/dL in the statin group. Baseline characteristics are summarized in Table 1. The statin group was more likely to be older and male, and have cardiovascular comorbidities than the no statin group. The prevalence of active cancer was not significantly different between the two groups. Types of surgery underwent are summarized Supplemental Table S1. The statin group also showed a higher incidence of postoperative coronary revascularization, but a lower incidence of ventilator care during in-hospital stay. At the time of hospital discharge, the prescription rates of cardiovascular medications other than statin were also higher in the statin group compared with the no statin group.

**Table 1:** Baseline characteristics.

	Entire population			IPTW		
	No statin (*n* = 3,778)	Statin (*n* = 1,331)	ASD	No statin (n = 4,053.1)	Statin (n = 1,067.8)	ASD
Peak postoperative troponin level, ng/L	1588 (± 14,853)	4,386 (± 28,861)	12.2	2,731 (± 17,881)	3,848 (± 24,027)	5.3
Preoperative statin treatment	685 (18.1)	1,142 (85.8)	> 99	1632.1 (40.3)	483.8 (45.3)	10.2
Male	2,183 (57.8)	849 (63.8)	12.3	2,459.2 (60.7)	630.0 (59.0)	3.4
Age	63.8 (± 14.6)	70.8 (± 9.8)	56.3	66.1 (± 13.9)	69.0 (± 10.7)	23.3
Diabetes	1937 (51.3)	809 (60.8)	19.2	2,215.9 (54.7)	639.2 (59.9)	10.5
Hypertension	2,277 (60.3)	1,146 (86.1)	61.0	2,828.0 (69.8)	890.0 (74.6)	18.7
Current smoking	327 (8.7)	126 (9.5)	2.8	339.0 (8.4)	125.2 (11.7)	11.2
Current alcohol	572 (15.1)	164 (12.3)	8.2	580.9 (14.3)	138.7 (13.0)	3.9
Chronic kidney disease	455 (12.0)	233 (17.5)	15.4	711.1 (17.5)	190.8 (17.9)	0.8
History of coronary artery disease	571 (15.1)	648 (48.7)	77.2	1,087.8 (29.3)	351.6 (32.9)	7.8
History of heart failure	122 (3.2)	73 (5.5)	11.1	162.7 (4.0)	55.7 (5.2)	5.7
History of stroke	294 (7.8)	168 (12.6)	16.0	476.8 (11.8)	135.9 (12.7)	2.9
History of arrhythmia	351 (9.3)	165 (12.4)	10.0	395.5 (9.8)	127.7 (12.0)	7.1
History of heart valve disease	76 (2.0)	30 (2.3)	1.7	74.6 (1.8)	14.7 (1.4)	3.7
Active cancer	1,500 (39.7)	490 (36.8)	5.9	1,580.4 (39.0)	333.2 (31.2)	16.4
**Operative variables**						
ESC/ESA surgical high risk	1,006 (26.6)	297 (22.3)	10.0	1,079.3 (25.7)	298.8 (28.0)	3.0
Operative duration, h	3.76 (± 2.94)	2.79 (± 1.92)	39.1	3.41 (± 2.73)	3.00 (± 1.92)	17.6
Emergency operation	1,043 (27.6)	240 (18.0)	23.0	964.8 (23.8)	341.3 (32.0)	18.3
General anesthesia	3,315 (87.7)	1,105 (83.0)	13.4	3,454.5 (85.2)	912.6 (85.5)	0.7
RBC transfusion	619 (16.4)	120 (9.0)	22.3	563.0 (13.9)	142.1 (13.3)	1.7
Continuous infusion of inotropics	1619 (42.9)	419 (31.5)	23.7	1523.8 (37.6)	405.7 (38.0)	0.8
**Postoperative in-hospital care**						
Coronary revascularization	44 (1.2)	122 (9.2)	36.8	362.3 (8.9)	48.4 (4.5)	17.7
Intensive care unit	2,831 (74.9)	933 (70.1)	10.8	3,048.8 (75.2)	803.7 (75.3)	0.1
ECMO	1 (0.0)	0	2.3	0.7 (0.0)	0	1.9
Continuous renal replacement therapy	54 (1.4)	9 (0.7)	7.4	48.6 (1.1)	6.8 (0.6)	5.2
Ventilator	999 (26.4)	209 (15.7)	26.6	910.4 (22.5)	228.8 (21.4)	2.5
**Discharge medication**						
Antiplatelet agent	863 (22.8)	788 (59.2)	79.6	1519.3 (37.5)	457.8 (42.9)	11.0
Beta blocker	637 (16.9)	540 (40.6)	54.3	1,045.7 (25.8)	279.2 (26.1)	0.8
Calcium-channel blocker	901 (23.8)	484 (36.4)	27.5	1,231.3 (30.4)	359.7 (33.7)	7.1
RAAS inhibitor	662 (17.5)	605 (45.5)	63.1	1,152.3 (28.4)	324.3 (30.4)	4.3
Direct oral anticoagulant	151 (4.0)	81 (6.1)	9.6	177.6 (4.4)	52.4 (4.9)	11.0
Warfarin	263 (7.0)	120 (9.0)	7.6	307.3 (7.6)	128.6 (12.0)	15.0

Data are presented as n (%) or mean (± standard deviation).

*IPTW* inversed probability treatment weighting, *ASD* absolute standardized difference, *ESC* European Society of cardiology, *ESA* European Society of Anaesthesiology, *ECMO* extracorporeal membranous oxygenation, *RAAS* renin–angiotensin–aldosterone system.

**Figure 2:**
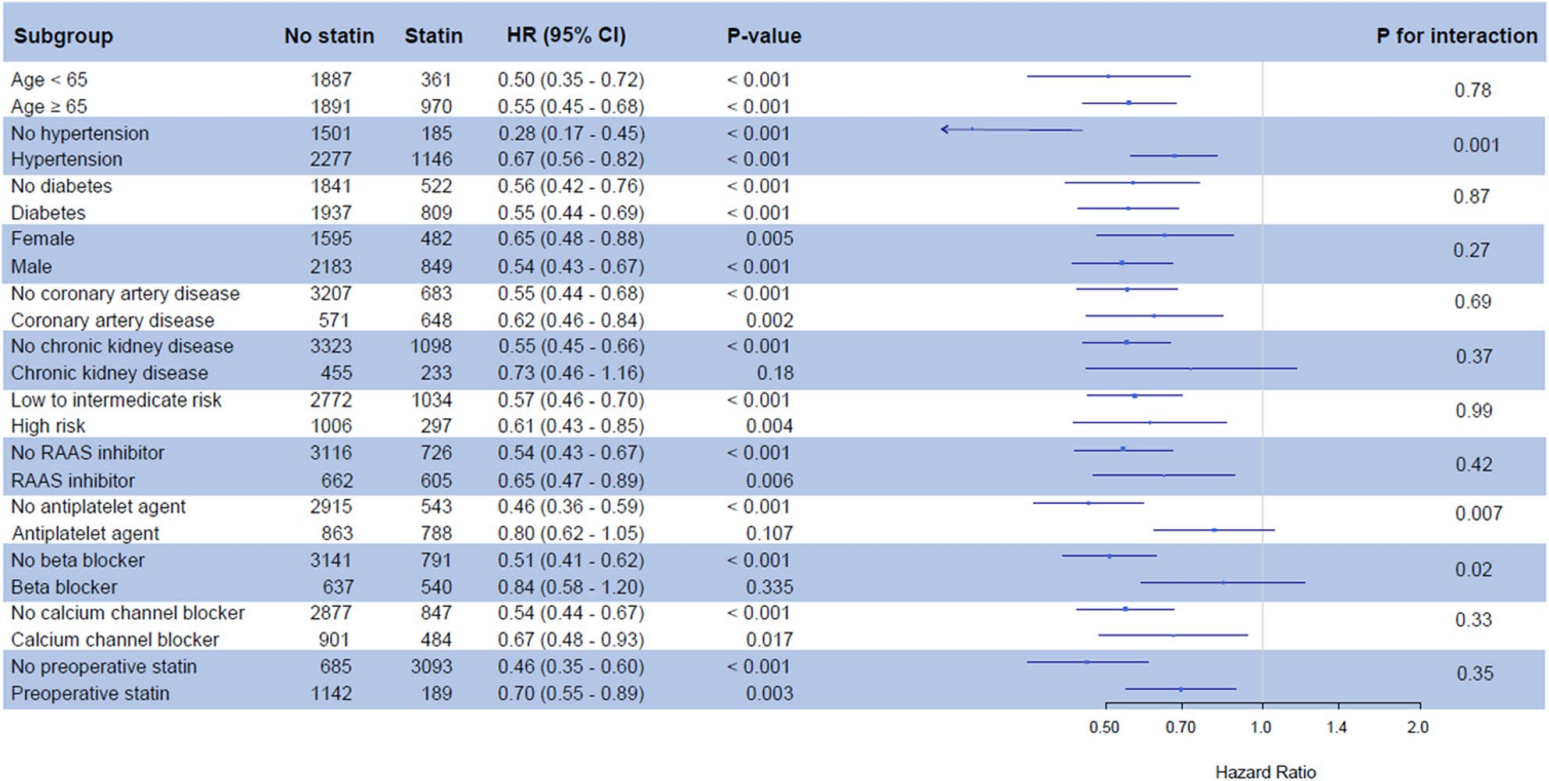
A forest plot for subgroup analysis.

In subgroup analysis, a significant interaction was observed with prescriptions of antiplatelet and beta blocker at discharge. This association may not be valid in patients with those prescriptions (HR, 0.80; 95% CI, 0.62–1.05; p = 0.107 in the patients with prescriptions of antiplatelet; p for interaction = 0.007 and HR, 0.84; 95% CI, 0.58–1.20; p = 0.335 in the patients with prescription of beta blocker; p for interaction = 0.020) (Fig. 2).

### Mortality after hospital discharge according to statin therapy

**Figure 3:**
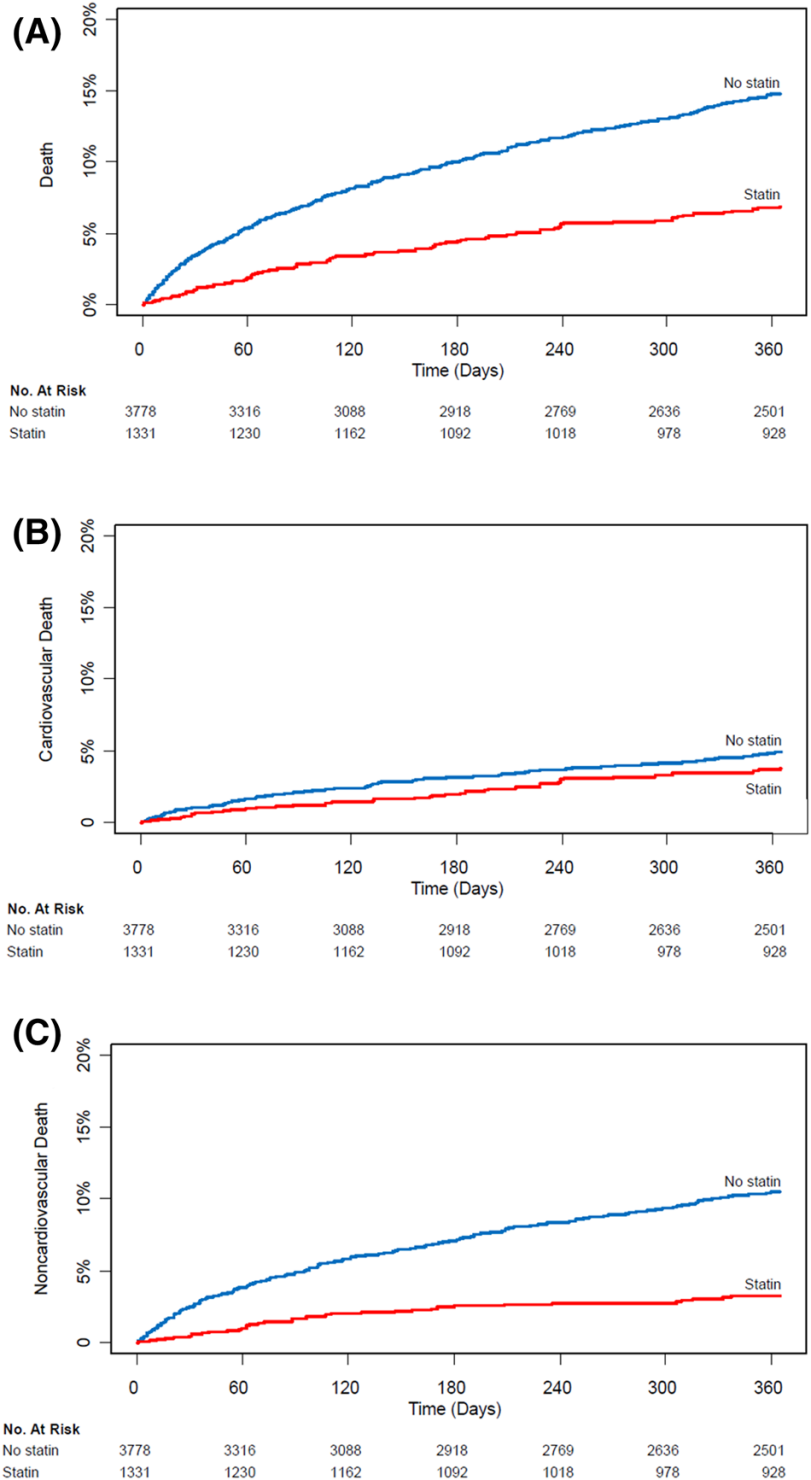
Kaplan–Meier curves 1-year mortality.

**Figure 4:**
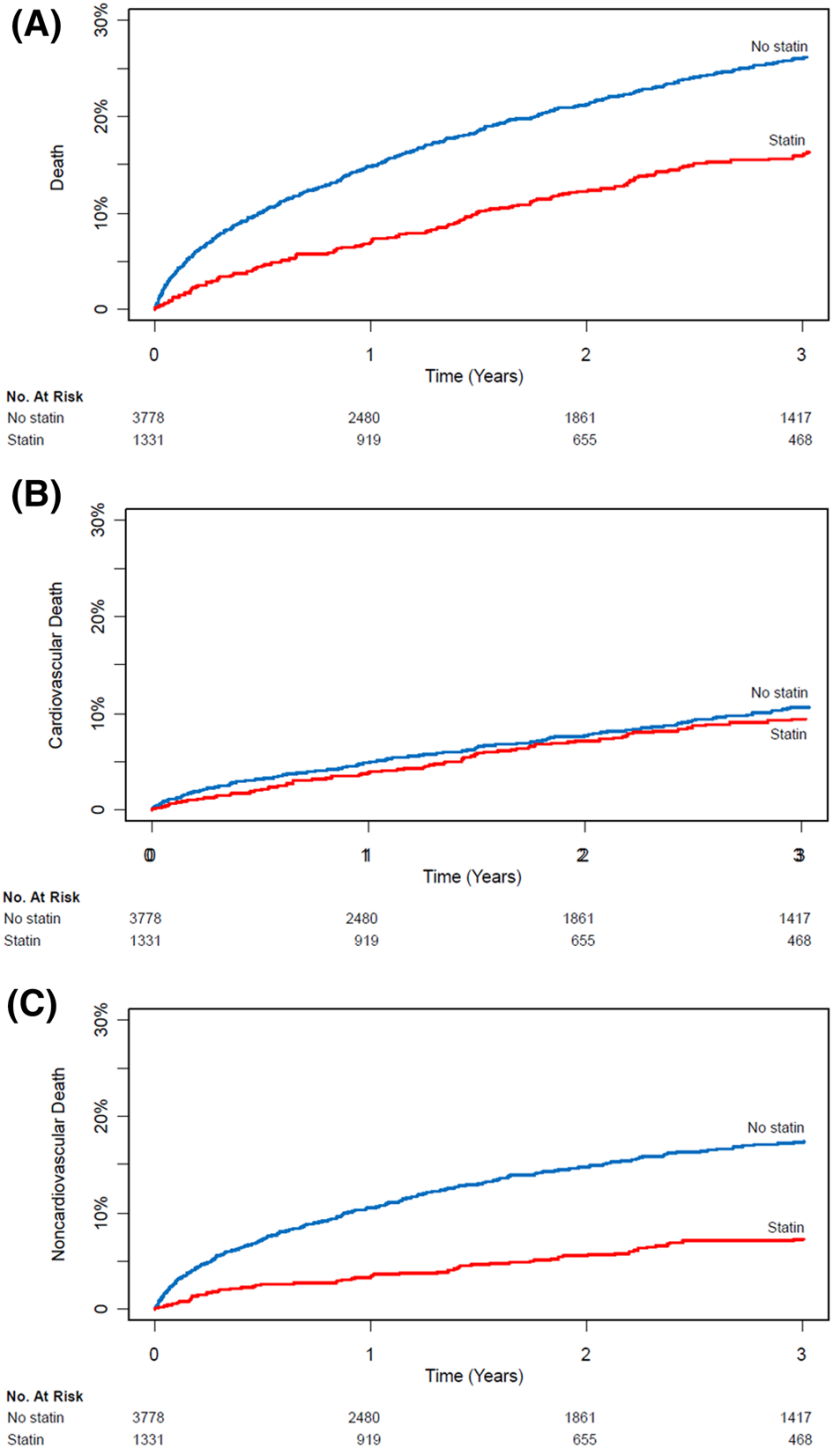
Kaplan–Meier curves for overall mortality.

The median follow-up durations of 1-year mortalities were 365 days (IQR 209–365) in the no statin group and 365 days (IQR 255–365) in the statin group. The 1-year mortality of the entire population was 11.4% (582/5,109). Before IPTW, the statin group had significantly lower risk of 1-year and overall mortalities compared with the no statin group (6.1% vs. 13.3%; adjusted HR, 0.55; 95% CI, 0.41–0.74; p < 0.001 for 1-year mortality and 15.0% vs. 25.0%; adjusted HR, 0.62; 95% CI, 0.51–0.76; p < 0.001 for overall mortality) (Table 2; Fig. 3). The corresponding results were consistent in IPTW analysis (adjusted HR, 0.61; 95% CI, 0.50–0.74; p < 0.001 for 1-year mortality and adjusted HR, 0.70; 95% CI, 0.54–0.90; p = 0.006 for overall mortality) (Table 2; Fig. 4). The lower risk of mortality in the statin group compared with the no statin group during 1-year follow-up was mainly driven by noncardiovascular deaths. Survival curves for overall and 1-year mortalities are shown in Fig. 3.

**Table 2:** Clinical outcomes of the entire population.

	Univariate analysis				Multivariate analysis		IPW analysis	
	No statin (*n* = 3,778)	Statin (*n* = 1,331)	Unadjusted HR (95% CI)	*P* value	*Adjusted HR (95% CI)	*P* value	**Adjusted HR (95% CI)	*P* value
One-year mortality	501 (13.3)	81 (6.1)	0.44 (0.35–0.55)	< 0.001	0.55 (0.41–0.74)	< 0.001	0.61 (0.50–0.74)	< 0.001
Cardiovascular	155 (4.1)	43 (3.2)	0.75 (0.54–1.05)	0.10	0.76 (0.49–1.18)	0.22	0.86 (0.67–1.12)	0.26
Noncardiovascular	346 (9.2)	38 (2.9)	0.30 (0.21–0.42)	< 0.001	0.42 (0.28–0.63)	< 0.001	0.43 (0.32–0.57)	< 0.001
Overall mortality	945 (25.0)	199 (15.0)	0.60 (0.51–0.70)	< 0.001	0.62 (0.51–0.76)	< 0.001	0.70 (0.54–0.90)	0.006
Cardiovascular	398 (10.5)	116 (8.7)	0.85 (0.69–1.05)	0.12	0.72 (0.55–0.94)	0.02	1.35 (0.94–1.94)	0.10
Noncardiovascular	547 (14.5)	83 (6.2)	0.42 (0.33–0.53)	< 0.001	0.53 (0.40–0.71)	< 0.001	0.39 (0.26–0.58)	< 0.001

Data are presented as n (%) or mean (± standard deviation).

*Covariates for multivariable analysis include preoperative statin treatment + age + male + diabetes + hypertension + ischemic heart disease + chronic kidney disease + heart failure + stroke + arrhythmia + surgical high risk + general anesthesia + emergency operation + operative duration + intraoperative red blood cell transfusion + intraoperative inotropic infusion + in-hospital coronary revascularization + in-hospital ventilator + in-hospital intensive care unit + antiplatelet agent + beta blocker + calcium channel blocker + renin–angiotensin–aldosterone system inhibitor.

**Multivariable analysis was further conducted in IPTW analysis after retaining preoperative statin treatment + age + diabetes + hypertension + smoking + active cancer + emergency operation + in-hospital coronary revascularization + antiplatelet agent + warfarin.

The dose of statin prescription is summarized in Supplemental Table S2, and the baseline characteristics of the patients according to the doses of statin are summarized in Supplemental Table S3. The clinical outcomes did not differ significantly by the dose of statin prescription (Table 3).

**Table 3:** Clinical outcomes according to the dose of statin.

	Low to moderate (n = 1,143)	High (n = 188)	Unadjusted HR (95% CI)	*P* value	*Adjusted HR (95% CI)	*P* value
One-year mortality	68 (5.9)	13 (6.9)	1.18 (0.65–2.14)	0.58	1.18 (0.64–2.19)	0.59
Cardiovascular	34 (3.0)	9 (4.8)	1.64 (0.79–3.42)	0.19	1.49 (0.68–3.24)	0.32
Noncardiovascular	34 (3.0)	4 (2.1)	0.73 (0.26–2.04)	0.54	1.30 (0.27–2.21)	0.62
Overall mortality	170 (14.9)	29 (15.4)	1.30 (0.87–1.93)	0.2	1.41 (0.93–2.12)	0.11
Cardiovascular	99 (8.7)	17 (9.0)	1.35 (0.81–2.27)	0.25	1.37 (0.79–2.35)	0.26
Noncardiovascular	71 (6.2)	12 (6.4)	1.23 (0.66–2.26)	0.52	1.42 (0.75–2.68)	0.28

Data are presented as n (%) or mean (± standard deviation).

*Covariates include age + male + diabetes + hypertension + smoking + smoking + ischemic heart disease + emergency operation + intraoperative red blood cell transfusion + in-hospital coronary revascularization + in-hospital ventilator care + in-hospital continuous renal replacement therapy + antiplatelet agent + beta blocker + calcium channel blocker + warfarin.

### Sensitivity analysis

Sensitivity of the effect of an unmeasured confounder on the observed association was computed assuming that the prevalence of measured confounder was 40%, and the association was significant under any circumstances (Supplemental Table S4). The hazard ratio and 95% CI for the primary outcome correspond with e-value of 3.26, indicating that the observed hazard ratio of 0.47 could be explained away by an unmeasured confounder that was associated with both statin prescription and mortality by a risk ratio of 3.26-fold each, above and beyond the measured confounders, but weaker confounding could not do so.

## Discussion

The results of this study showed that statin prescription at discharge was independently associated with improved mortality after MINS. A significant interaction was observed with prescriptions of antiplatelet and beta blocker at discharge, and the protective effect of statin use was not distinct in patients with those prescriptions. With the other previous studies showing a beneficial effect of statin use for patients diagnosed with MINS^3,12^, our findings suggest that statin prescription at discharge may be beneficial for patients who suffered MINS during the hospital stay.

MINS is a strong predictor of postoperative mortality up to 2 years after surgery^2,13^. Diagnostic criteria of MINS include at least one value of cardiac troponin elevation above the 99th percentile upper reference limit within 30 days after surgery, as a result of myocardial ischemia without the requirement of an ischemic symptom, based on clinical relevance shown in previous studies^1,12,14–16^. In these studies, the occurrence of MINS was associated with a four-fold increased risk of 30-day mortality regardless of symptoms^12^, and even an asymptomatic cardiac troponin elevation was associated with a three-fold increased risk for 30-day mortality^15^. Despite the increasing evidences of adverse outcomes after MINS, little is known about the treatment for these patients.

Recently, a randomized controlled trial demonstrated that direct oral anticoagulant effectively lowers cardiovascular complications in MINS patients, highlighting the risk of major thrombotic complications and supporting the need of secondary prevention^17^. Statin has also been widely used for primary and secondary prevention of atherosclerotic disease with lipid lowering effect^6^. In previous studies, the immediate use of statin after surgery in combination with other cardiovascular medications was significantly associated with improved outcomes in MINS patients^3^. Based on these studies, the one perioperative guideline regarding the management of MINS patients recommended the use of aspirin and statin^18^. However, the exact mechanism remains unknown.

Interesting finding in our study is that the reduced mortality in the statin group was mainly driven by the decreased noncardiovascular deaths. According to the VISION (The Vascular events In noncardiac Surgery patIents cOhort evaluation) study, the largest dedicated registry, more than half of deaths were related to vascular causes in the early phase of MINS^19^, but we rather focused on the long-term follow-up of the patients who were discharged alive after MINS. One previous study using nationwide database reported that survivors of perioperative myocardial infarction had a high rate of hospital readmission and 75% of such rehospitalization were due to noncardiovascular causes such as infection, bleeding, gastrointestinal, or pulmonary complications. In the long-term follow-up, statin offers clinical benefits by changing endothelial function and decreasing inflammation, which is known as the pleiotropic effect beyond the lipid lowering effect^7,20–22^. Moreover, statin has been suggested to have potential survival benefit for malignancies^23^, which comprises one of the main cause of noncardiac surgical mortality. Considering that surgical patients frequently encounter pathologic conditions that are associated with troponin elevation other than coronary cause^2^, the protective effect of statin in MINS patients might not be solely from the lipid lowering effect.

The overall protective effect of perioperative statin has shown inconsistent results among the relevant studies, and therefore remains an area of clinical equipoise^21^. The controversy has recently shifted as to whether there are particular conditions that perioperative statin use could be beneficial. The results of our study provides a clue for this question, suggesting statin use for secondary prevention of further adverse events in patients who already had MINS rather than primary prevention of MINS. And also, this study has strength that it covers a broad range of almost all types of noncardiac surgery and has clinical implications on a large population, as 8 million patients have been reported with MINS out of more than 200 million noncardiac surgery patients every year^24^. Moreover, the risk of cardiac complications is increasing as the average age of patients is increasingly older^25^. However, further studies are needed to establish causality of our results and determine the optimal timing, duration, and dose of statin use^21^.

To test generalizability of the observed association, we conducted a subgroup analysis, and showed that the beneficial effects of statin significantly interacted with the prescriptions of other medication. Additionally, we used two different statistical methods to conduct sensitivity analysis. Although our results were consistently significant in the method adjusting with random unmeasured confounder^26^, the calculated e-value was 3.26^27^. Calculating the e-value is another statistical method that represents robustness of the association regarding the potential of unmeasured confounders. The lowest value is 1.0, and the higher the value, the stronger the impact of unmeasured confounders.

This study should be appraised considering the following limitations. First, as an observational study, our results might have been affected by confounding factors. Despite rigorous statistical adjustments, unmeasured variables could not be corrected for. Second, perioperative hs-cTn was not a routine evaluation but selectively measured at our institution. Despite the institutional protocol, the possibility of selection bias exists. Third, dose, timing, and indications of statin therapy have not been considered, and lipid profile was not routinely checked per protocol in every patient. Moreover, this study was conducted in patients from a single center, and our result might not be generalized in other centers. Considering these limitations and the lack of causality establishment, the observed association in this study may not be robust enough to change the daily practice, and a well-designed study with a detailed protocol for hs-cTn measurement and statin use is needed. However, this is the first study to show the association between statin prescription at discharge and long-term survival benefit in patients suffering MINS, providing a valuable information for future hypothesis generation and trial design.

## Methods

The Institutional Review Board at Samsung Medical Center approved this observational study and waived the requirement for written informed consent for access to the registry (SMC 2019-08-048), because the entire data were in de-identified form from the initial curation. This study was conducted according to the principles of the Declaration of Helsinki and reported according to the Strengthening the Reporting of Observational studies in Epidemiology guideline (Supplemental Table S5).

### Study population and data curation

The data for this study were extracted from SMC-TINCO registry (Samsung Medical Center Troponin in Noncardiac Operation, KCT0004244). It is a large single-center de-identified cohort. It consists of 43,019 consecutive patients, from January 2010 and June 2019, who had at least one measured high-sensitivity cardiac troponin (hs-cTn) I before or within 30 days after noncardiac surgery in our institution. We operate a paperless hospital with electronic medical record system. It contains data of more than 4 million patients with more than 2 million surgeries, 900 million laboratory findings, and 200 million prescriptions. The data for this registry were entirely extracted using the “Clinical Data Warehouse Darwin-C” of our institution, which was built for investigators to search and retrieve de-identified medical records from this electronic archive system. The mortalities in this system are consistently updated and confirmed by the National Population Registry of the National Statistical Office using a unique personal identification number, when available. Baseline characteristics of the patients were organized based on the automatically extracted preoperative evaluation sheets by independent investigators who were blinded to hs-cTn levels and mortalities.

From the registry, we excluded the patients who were younger than 18 years at the time of surgery, the patients without postoperative hs-cTn I measurements or elevation, the patients with hs-cTn elevation from other than ischemic cause, and the patients with mortality during the hospital stay. Finally, the patients who were discharged after suffering MINS during the hospital stay were identified and were divided into two groups according to statin prescription.

### Definitions and study endpoints

MINS was initially identified by peak postoperative hs-cTn elevation above the 99th percentile upper reference limit which was 40 ng/L using an automated analyzer (Advia Centaur XP, Siemens Healthcare Diagnostics, Erlangen, Germany) with highly sensitive immunoassay within 30 days after surgery, and an elevation with an evidence of non-ischemic etiology such as sepsis, pulmonary embolus, atrial fibrillation, cardioversion, or chronic elevation was excluded from the diagnosis of MINS following the recent diagnostic criteria^14^. Past medical history was organized by reviewing the extracted electronic medical records. Active cancer was defined as histologic diagnosis of cancer within the previous 6 months^28^. High-risk surgery was defined according to the 2014 European Society of Cardiology/Anesthesiology (ESC/ESA) guidelines^29^. The dose of statin was defined as high when ≥ 20 mg of rosuvastatin or 40 mg of atorvastatin, and all lower doses were categorized as low-to moderate-dose.

The primary endpoint was 1-year mortality after discharge, and all-cause mortality during the overall follow-up period was also compared. Mortality was classified into cardiovascular and noncardiovascular mortality. Cardiovascular mortality was defined as death due to myocardial infarction, cardiac arrhythmia, heart failure, stroke, vascular causes. All deaths without an undisputed noncardiovascular cause were considered cardiovascular death. Noncardiovascular mortality was defined as death from a cause other than cardiovascular condition^30^.

### Perioperative management and hs-cTn I

Perioperative managements followed the institutional protocols which were based on current guidelines. Perioperative period hs-cTn I was selectively measured in patients undergoing moderate- or high-risk surgery or those with at least one of the major cardiovascular risk factors such as a history of ischemic heart disease, heart failure, stroke including transient ischemic attack, diabetes mellitus on insulin therapy, or chronic kidney disease based on current guidelines^29^, and in patients with mild risk, it was measured at the discretion of attending clinician.

### Statistical analysis

To evaluate the hypothesis, the statistical analysis was conducted by the two statistical experts at our institutional biostatistics center as planned before a formal analysis, and no further analysis was added according to the result of primary analysis. Baseline characteristics are presented as numbers and percentages for categorical variables and mean ± standard deviation (SD) or median with interquartile range (IQR) for continuous variables. Differences between each group were compared using Chi-square or Fisher’s exact test for categorical variables and the *t* test or the Mann–Whitney test for continuous variables. Kaplan–Meier survival curves were constructed and compared with the log-rank test. To retain a large sample size and maximize the study power while maintaining a balance in covariates between the 2 groups, we conducted rigorous adjustment for differences in baseline and lesion characteristics of patients using the weighted Cox proportional-hazards regression models with the inverse probability of treatment weighting (IPTW). According to this technique, weights for patients without statin treatment were the inverse of the propensity score and weights for patients receiving statin were the inverse of 1 − the propensity score^31^. The reductions in mortalities were compared using a stratified Cox regression model. After IPTW, covariates with a standardized mean difference > 0.1 were retained in the multivariable model to report adjusted hazard ratios (HRs) with 95% confidence intervals (CIs). We also performed subgroup analysis using IPTW to reveal hidden interactions.

For sensitivity analysis, we estimated the potential impact of unmeasured confounders and calculated e-values which represent the strength of association on the risk ratio scale that an unmeasured confounder would need to have with both statin prescription and the change/interruption of third component, conditional on the measured covariates, to explain away the observed association^26,27^ Spearman's rank correlation was used to evaluate the power of this study regarding the sample size^32^, and the power of analysis after IPTW adjustment was 0.99 when HR was lower than 0.7 and 0.90 when HR was 0.8. All statistical analyses were performed with R 3.5.3 (Vienna, Austria; https://www.R-project.org/). All tests were 2-tailed, and p < 0.05 was considered statistically significant.

## Conclusion

In patients who had MINS, statin prescription at discharge was associated with reduced long-term mortality. A randomized trial is needed to confirm this finding.

## Supplementary information

Supplementary tables.
