# Supplementary material for: Postoperative statin treatment may be associated with improved mortality in patients with myocardial injury after noncardiac surgery
Source: Sci Rep. 2020 Jul 15;10:11616. doi: 10.1038/s41598-020-68511-3 (PMC7363808; doi:10.1038/s41598-020-68511-3)
Supplement: Supplementary file 1 — Supplementary tables. [file 41598_2020_68511_MOESM1_ESM.docx]

**Postoperative Statin Treatment May be Associated With Improved Mortality in Patients With Myocardial Injury After Noncardiac Surgery**

Jungchan Park, MD^1^, Jihoon Kim, MD^2^, Seung-Hwa Lee, MD^2^, Jong Hwan Lee, MD, PhD^1^, Jeong Jin Min, MD^1^, Ji-hye Kwon MD^1^, Ah Ran Oh, MD^1^, Wonho Seo, MD^1^, Cheol Won Hyeon, MD^2^ , Kwangmo Yang, MD^3^, Jin-ho Choi, MD, PhD, Sang-Chol Lee, MD, PhD^2^, Kyunga Kim, PhD^4,5^, Joonghyun Ahn^4^, and Hyeon‐Cheol Gwon, MD, PhD^2^

^1^Department of Anesthesiology and Pain Medicine, Samsung Medical Center, Sungkyunkwan University School of Medicine, Seoul, Korea

^2^Division of Cardiology, Department of Medicine, Heart Vascular Stroke Institute, Samsung Medical Center, Sungkyunkwan University School of Medicine, Seoul, Korea

^3^Centers for Health Promotion, Samsung Medical Center, Sungkyunkwan University School of Medicine, Seoul, Korea

^4^Statistics and Data Center, Research Institute for Future Medicine, Samsung Medical Center, Seoul, Korea

^5^Department of Digital Health, SAIHST, Sungkyunkwan University, Seoul, Korea

The first two authors contributed equally to this work (J.Park, J.Kim).

Running title: Statins in patients with myocardial injury after noncardiac surgery

Supplemental table 1. Types and doses of statin

| Type | Dose | n |
| --- | --- | --- |
| Atorvastatin | 10 mg | 448 |
|  | 20 mg | 200 |
|  | 40 mg | 76 |
|  | 80 mg | 20 |
| Rosuvastatin | 5 mg | 23 |
|  | 10 mg | 235 |
|  | 20 mg | 92 |
| Simvastatin | 20 mg | 86 |
|  | 40 mg | 7 |
| Pitavastatin | 2 mg | 79 |
|  | 4 mg | 4 |
| Fluvastatin | 80 mg | 19 |
| Pravastatin | 10 mg | 16 |
|  | 20 mg | 12 |
|  | 40 mg | 14 |

Supplemental table 2. Types of surgery

|  | No statin | Statin |
| --- | --- | --- |
|  | (n=3778) | (n=1331) |
| Vascular | 234 (6.2) | 307 (23.1) |
| Orthopediatric | 525 (13.9) | 263 (19.8) |
| Neuro | 467 (12.4) | 117 (8.8) |
| Breast or Endo | 79 (2.1) | 16 (1.2) |
| Plastic or Otolaryngeal or Eye | 142 (3.8) | 41 (3.1) |
| Transplantation | 456 (12.1) | 12 (0.9) |
| Gynecology or Urology | 322 (8.5) | 85 (6.4) |
| Gastrointestinal | 993 (26.3) | 225 (16.9) |
| Noncardiac thoracic | 541 (14.3) | 256 (19.2) |
| Others | 19 (0.5) | 9 (0.7) |

Supplemental table 3. Baseline characteristics according to the intensity of statin

|  | Entire population | | |
| --- | --- | --- | --- |
|  | Low to moderate | High | P-value |
|  | (n = 1143) | (n = 188) |  |
| Male | 711 (62.2) | 138 (73.4) | 0.004 |
| Age | 71.1 (±9.7) | 68.8 (±9.9) | 0.003 |
| Diabetes | 683 (59.8) | 126 (67.0) | 0.07 |
| Hypertension | 991 (86.7) | 155 (82.4) | 0.15 |
| Current smoking | 101 (8.8) | 25 (13.3) | 0.07 |
| Current alcohol | 141 (12.3) | 23 (12.2) | >0.99 |
| Chronic kidney disease | 196 (17.1) | 37 (19.7) | 0.46 |
| History of coronary artery disease | 540 (47.2) | 108 (57.4) | 0.01 |
| History of heart failure | 62 (5.4) | 11 (5.9) | 0.95 |
| History of stroke | 143 (12.5) | 25 (13.3) | 0.86 |
| History of arrhythmia | 146 (12.8) | 19 (10.1) | 0.36 |
| History of heart valve disease | 27 (2.4) | 3 (1.6) | 0.7 |
| Active cancer | 425 (37.2) | 65 (34.6) | 0.55 |
| Operative variables |  |  |  |
| ESC/ESA surgical high risk | 250 (21.9) | 47 (25.0) | 0.39 |
| Operative duration, hours | 2.77 (±1.93) | 2.90 (±1.88) | 0.38 |
| Emergency operation | 214 (18.7) | 26 (13.8) | 0.13 |
| General anesthesia | 951 (83.2) | 154 (81.9) | 0.74 |
| RBC transfusion | 100 (8.7) | 20 (10.6) | 0.48 |
| Continuous infusion of inotropics | 355 (31.1) | 64 (34.0) | 0.46 |
| Postoperative in-hospital care |  |  |  |
| Coronary revascularization | 77 (6.7) | 45 (23.9) | <0.001 |
| Intensive care unit | 794 (69.5) | 139 (73.9) | 0.25 |
| ECMO | 0 | 0 |  |
| Continuous renal replacement therapy | 6 (0.5) | 3 (1.6) | 0.24 |
| Ventilator | 173 (15.1) | 36 (19.1) | 0.2 |
| Discharge medication |  |  |  |
| Antiplatelet agent | 655 (57.3) | 133 (70.7) | 0.001 |
| Beta blocker | 446 (39.0) | 94 (50.0) | 0.01 |
| Calcium-channel blocker | 429 (37.5) | 55 (29.3) | 0.04 |
| RAAS inhibitor | 524 (45.8) | 81 (43.1) | 0.53 |
| Direct oral anticoagulant | 66 (5.8) | 15 (8.0) | 0.31 |
| Warfarin | 109 (9.5) | 11 (5.9) | 0.13 |

Data are presented as n (%) or mean (±standard deviation)

ESC, European Society of cardiology; ESA, European Society of Anaesthesiology; ECMO, extracorporeal membranous oxygenation; RAAS, renin-angiotensin-aldosterone system

Supplemental table 4. Sensitivity analysis of the effect of an unmeasured confounder on hazard ratio of statin for mortality after myocardial injury after noncardiac surgery

|  |  | OR*_ZY_*_\|_*_X_* | | | | | |
| --- | --- | --- | --- | --- | --- | --- | --- |
|  |  | 1.5 | 2 | 2.5 | 3 | 3.5 | 4 |
| OR_zx_ | 0.3 | 0.56 (0.47-0.65) | 0.59 (0.51-0.70) | 0.63 (0.54-0.74) | 0.65 (0.55-0.76) | 0.69 (0.59-0.81) | 0.69 (0.59-0.81) |
|  | 0.4 | 0.55 (0.47-0.65) | 0.57 (0.49-0.67) | 0.61 (0.52-0.71) | 0.63 (0.54-0.74) | 0.64 (0.55-0.75) | 0.66 (0.57-0.78) |
|  | 0.5 | 0.54 (0.46-0.63) | 0.57 (0.48-0.66) | 0.60 (0.51-0.70) | 0.60 (0.51-0.70) | 0.61 (0.52-0.72) | 0.63 (0.54-0.74) |
|  | 0.6 | 0.54 (0.46-0.63) | 0.56 (0.47-0.65) | 0.57 (0.49-0.67) | 0.57 (0.49-0.67) | 0.58 (0.50-0.68) | 0.59 (0.51-0.69) |
|  | 0.7 | 0.54 (0.46-0.63) | 0.54 (0.46-0.63) | 0.56 (0.48-0.65) | 0.55 (0.47-0.64) | 0.56 (0.48-0.65) | 0.58 (0.49-0.68) |

Prevalence of unmeasured confounder = 40%

Numbers represent HRs (including 95% CIs).

OR, odds ratio; HR, hazard ratio; X: dichotomous exposure measure, y dichotomous outcome measure, z : potential dichotomous confounder.

ORzx indicates the association (OR) between the unmeasured confounder and statin.

OR*_ZY_*_|_*_X_* indicates the association between the unmeasured confounder and mortality.

Supplemental table 5. Strengthening the Reporting of Observational studies in Epidemiology guideline Statement—Checklist of items that should be included in reports of cohort studies

|  | | Item No | Recommendation | Page No |
| --- | --- | --- | --- | --- |
| **Title and abstract** | | 1 | (*a*) Indicate the study’s design with a commonly used term in the title or the abstract | 1 |
|  |  |  | (*b*) Provide in the abstract an informative and balanced summary of what was done and what was found | 1 |
| Introduction | | | | |
| Background/rationale | | 2 | Explain the scientific background and rationale for the investigation being reported | 4 |
| Objectives | | 3 | State specific objectives, including any prespecified hypotheses | 4 |
| Methods | | | | |
| Study design | | 4 | Present key elements of study design early in the paper | 10 |
| Setting | | 5 | Describe the setting, locations, and relevant dates, including periods of recruitment, exposure, follow-up, and data collection | 11 |
| Participants | | 6 | (*a*) Give the eligibility criteria, and the sources and methods of selection of participants. Describe methods of follow-up | 11 |
|  |  |  | (*b*) For matched studies, give matching criteria and number of exposed and unexposed |  |
| Variables | | 7 | Clearly define all outcomes, exposures, predictors, potential confounders, and effect modifiers. Give diagnostic criteria, if applicable | 12 |
| Data sources/ measurement | | 8* | For each variable of interest, give sources of data and details of methods of assessment (measurement). Describe comparability of assessment methods if there is more than one group | 11-12 |
| Bias | | 9 | Describe any efforts to address potential sources of bias | 13 |
| Study size | | 10 | Explain how the study size was arrived at | 14 |
| Quantitative variables | | 11 | Explain how quantitative variables were handled in the analyses. If applicable, describe which groupings were chosen and why | 14 |
| Statistical methods | | 12 | (*a*) Describe all statistical methods, including those used to control for confounding | 14 |
|  |  |  | (*b*) Describe any methods used to examine subgroups and interactions |  |
|  |  |  | (*c*) Explain how missing data were addressed |  |
|  |  |  | (*d*) If applicable, explain how loss to follow-up was addressed |  |
|  |  |  | (*e*) Describe any sensitivity analyses |  |
| Results | | | |  |
| Participants | | 13* | (a) Report numbers of individuals at each stage of study—eg numbers potentially eligible, examined for eligibility, confirmed eligible, included in the study, completing follow-up, and analysed | 5 |
|  |  |  | (b) Give reasons for non-participation at each stage |  |
|  |  |  | (c) Consider use of a flow diagram |  |
| Descriptive data | | 14* | (a) Give characteristics of study participants (eg demographic, clinical, social) and information on exposures and potential confounders | 5 |
|  |  |  | (b) Indicate number of participants with missing data for each variable of interest |  |
|  |  |  | (c) Summarise follow-up time (eg, average and total amount) |  |
| Outcome data | | 15* | Report numbers of outcome events or summary measures over time |  |
| Main results | 16 | (*a*) Give unadjusted estimates and, if applicable, confounder-adjusted estimates and their precision (eg, 95% confidence interval). Make clear which confounders were adjusted for and why they were included | | 6 |
|  |  | (*b*) Report category boundaries when continuous variables were categorized | |  |
|  |  | (*c*) If relevant, consider translating estimates of relative risk into absolute risk for a meaningful time period | |  |
| Other analyses | 17 | Report other analyses done—eg analyses of subgroups and interactions, and sensitivity analyses | | 6 |
| Discussion | | | | |
| Key results | 18 | Summarise key results with reference to study objectives | | 6 |
| Limitations | 19 | Discuss limitations of the study, taking into account sources of potential bias or imprecision. Discuss both direction and magnitude of any potential bias | | 9 |
| Interpretation | 20 | Give a cautious overall interpretation of results considering objectives, limitations, multiplicity of analyses, results from similar studies, and other relevant evidence | | 7 |
| Generalisability | 21 | Discuss the generalisability (external validity) of the study results | | 9 |
| Other information | | | | |
| Funding | 22 | Give the source of funding and the role of the funders for the present study and, if applicable, for the original study on which the present article is based | | 2 |
